# Vertebral Fracture Associated With Extracorporeal Shock Wave Lithotripsy: A Case Report

**DOI:** 10.7759/cureus.57900

**Published:** 2024-04-09

**Authors:** Ahmed A Al Bazroon, Ahmed A Albassri, Tarek Swellam, Ibrahim Al Basha

**Affiliations:** 1 Department of Urology, Dammam Medical Complex, Dammam, SAU; 2 Department of Radiology, Dammam Medical Complex, Dammam, SAU

**Keywords:** eswl (extracorporeal shockwave lithotripsy), renal stone case report, ureteric stone, vertebral fracture, eswl complications

## Abstract

Extracorporeal shock wave lithotripsy (ESWL) is considered a safe, reliable, and non-invasive modality for kidney stone management. However, there are well-established complications related to ESWL documented in the literature in the form of renal and extrarenal complications. Skeletal complications related to ESWL are rarely recorded; as far as we know, there is only one documented case report of an ESWL-related burst vertebral fracture seen in an osteoporotic patient, diagnosed as granulomatous spondylitis. Here, we present a novel case of a transverse process fracture of the third lumbar vertebra related to ESWL in a young patient otherwise free from any medical illness.

## Introduction

Skeletal complications related to extracorporeal shock wave lithotripsy (ESWL) are poorly documented in the literature; however, the use of ESWL to manage some of the orthopedic diseases like pseudoarthrosis and tendinopathy has been documented in the literature [1,2]. Since its introduction in the 1980s, ESWL has brought revolutionary changes in urolithiasis management [3]. Despite the non-invasive nature of ESWL, there are well-documented ESWL-related complications such as perirenal, subcapsular and intrarenal hematomas [4], and some extrarenal complications such as gastrointestinal complications [5], infection-related complications [6] and fertility-related complications [7,8]. However, it is unknown if ESWL also has skeletal-related complications. To our knowledge, there is only one case reported in the literature regarding an ESWL-related burst vertebral fracture seen in an old osteoporotic man, diagnosed later on as granulomatous spondylitis [9]. Here, we present a novel case of a transverse process fracture of the third lumbar vertebra following ESWL in a 40-year-old man not suffering from any other medical illness.

## Case presentation

**Figure 1:**
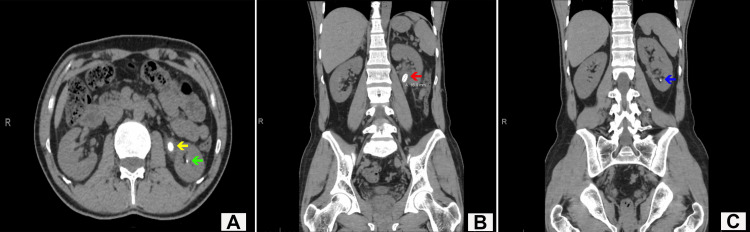
(A) Axial view of CT KUB showing a large renal pelvis stone (yellow arrow) and a small lower pole calyceal stone (green arrow). (B) Coronal view of CT KUB showing the large renal pelvis stone (red arrow). (C) Coronal view of CT KUB showing a small, non-obstructive lower pole calyceal stone (blue arrow). KUB: kidneys, ureters and bladder

A 40-year-old man, who was a known stone-former patient, presented to the urology clinic with a history of intermittent mild-to-moderate left flank pain that radiated to the ipsilateral groin. It was associated with dysuria, frequency and urgency. He had no history of hematuria, fever, nausea, anorexia or vomiting, or illicit drug usage. Initial plain abdominal computed tomography (CT) showed a large stone in the renal pelvis measuring 1.3 × 0.9 × 1.7 cm with mild focal dilatation of the renal pelvis with another 3-mm non-obstructive, lower pole calyceal stone seen. The stone Hounsfield unit (HU) value was 1100 (Figures 1A-1C).

**Figure 2:**
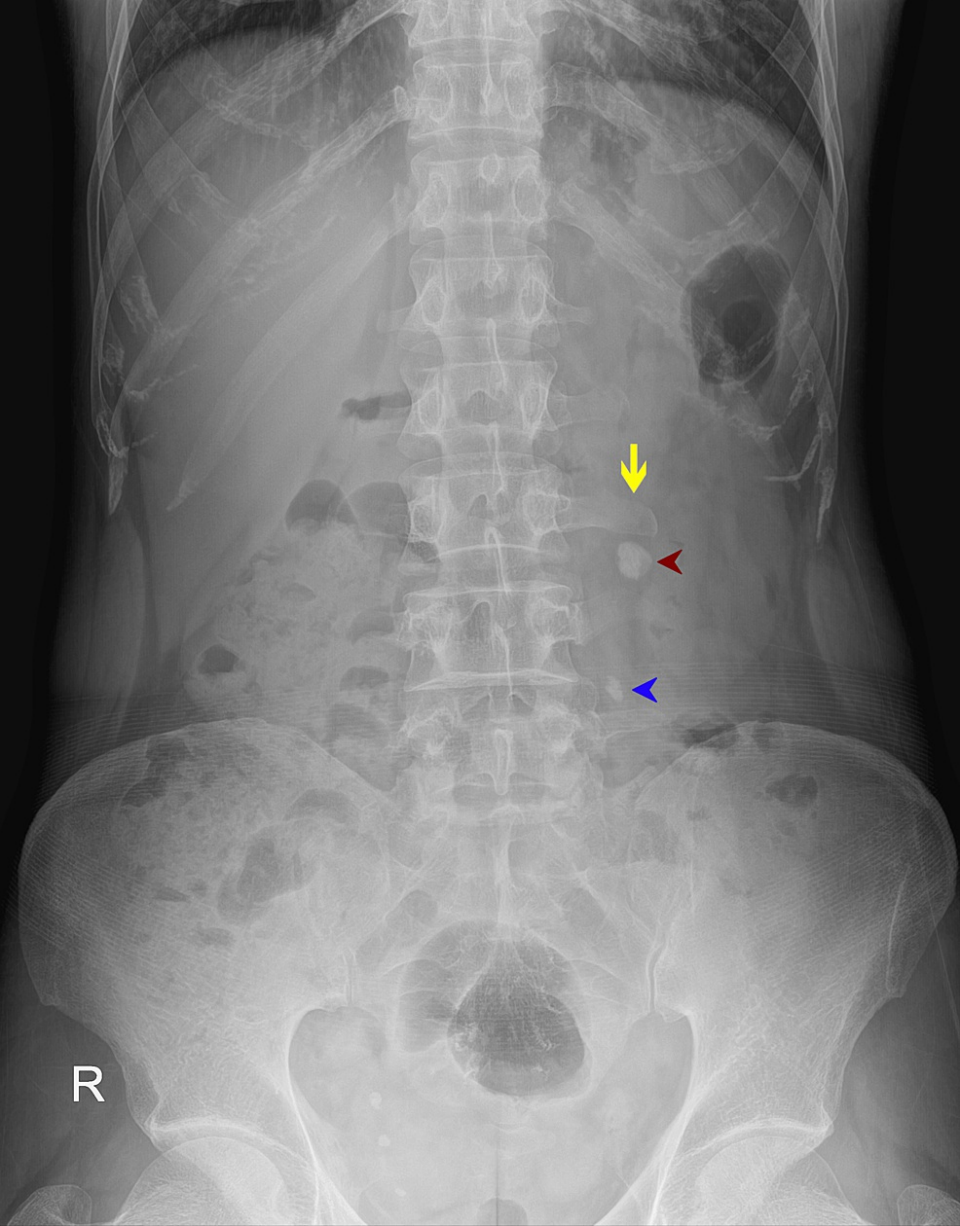
Initial X-ray KUB that was done prior to ESWL showing a normal, intact left transverse process of L3 vertebrae (yellow arrow). Radiopaque shadow of the large left renal pelvis stone (red arrowhead) and radiopaque shadow of the left upper ureteric stone (blue arrowhead) are seen KUB: kidneys, ureters and bladder, ESWL: extracorporeal shock wave lithotripsy

Initial labs showed normal creatinine and urea levels, and no leukocytosis. As the patient had a previous history of renal stones treated and cleared by ESWL, we offered to treat him by ESWL given the good response for previous stones, and he agreed with the treatment plan. The initial X-ray of the kidneys, ureters and bladder (KUB) showed a radiopaque large renal pelvis stone and a proximal left ureteric stone (Figure 2).

**Figure 3:**
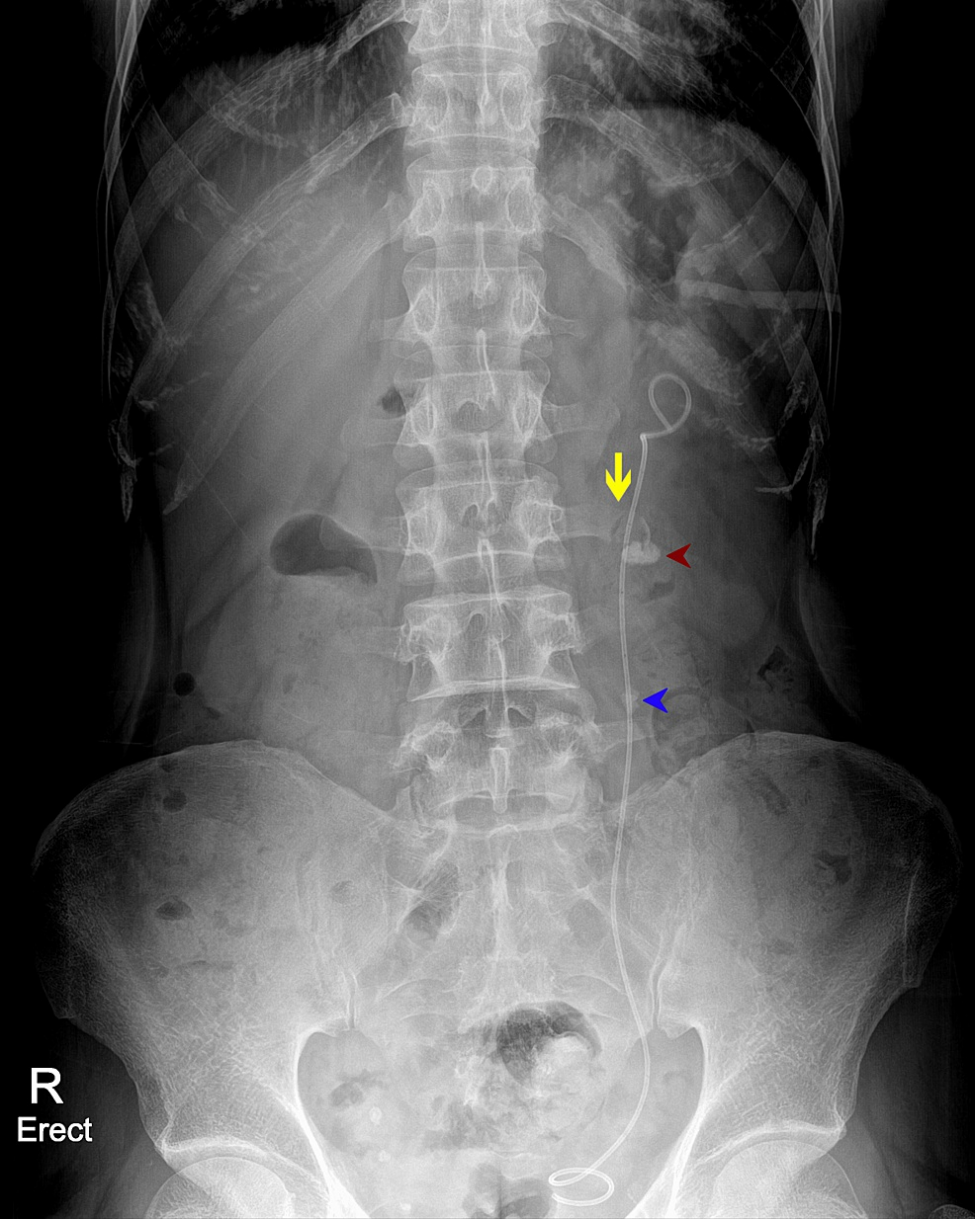
X-ray KUB that was done after the last session of ESWL showing a longitudinal fissure fracture of the left transverse process of L3 vertebrae (yellow arrow). Radiopaque shadow of the partially fragmented left renal pelvis stone (red arrowhead) and radiopaque shadow of the partially fragmented left upper ureteric stone (blue arrowhead) are seen KUB: kidneys, ureters and bladder, ESWL: extracorporeal shock wave lithotripsy

The Dornier Gemini (EMSE 220f-XXP) electromagnetic shock wave lithotripter was used for lithotripsy in a total of 14.278 shock waves delivered in five sessions, one session per month over a period of five months. He was kept on tamsulosin 0.4 mg daily. He had a good response to ESWL and passed multiple gravels after each session. Stone analysis revealed that the stone was composed of calcium oxalate. After the third session of ESWL, the patient had an emergency insertion of a left (LT) ureteric JJ stent as he had persistent colic. The remaining two sessions of ESWL were done subsequently. The patient came for regular clinic visits; KUB X-ray after the fifth session showed partial fragmentation of the upper ureteric stone, and a clear longitudinal fissure fracture of the transverse process of the third lumbar vertebra was appreciated (Figure 3).

Subsequent history was taken and the patient denied any history of trauma. The patient had only mild back and left flank pain. Neurosurgery and orthopedic surgery consultations were done, and it was decided that such a stable in-place fissure fracture did not need further intervention. Bed rest and analgesics were recommended accordingly. Patient’s pain improved with treatment, he became pain-free and no further imaging was done for the fracture. The patient did well and he electively underwent laser lithotripsy with ureteroscopy, which resulted in complete stone fragmentation, and an LT ureteric JJ stent was placed. The stent was removed four weeks later, and the patient was discharged in a good condition.

## Discussion

Since its introduction in the 1980s, ESWL has become the standard, convenient, most common non-invasive procedure for treating renal and ureteric calculi [2]. ESWL is a highly effective and relatively safe minor procedure with a severe life-threatening complication rate of <1% [10]. ESWL generates high-energy shock waves that cause stone fragmentation via direct stress and cavitation [11]. Many well-established ESWL-related complications, either renal or extrarenal, have been documented. For better outcomes, many strategies, such as giving slower shock wave rates and following a stepwise protocol, have been suggested, which cause stone fragmentation at a lower complication rate [12,13]. However, ESWL-related skeletal complications are poorly investigated in the literature, and to our knowledge, there is only one case of a vertebral fracture seen in an osteoporotic patient who underwent ESWL for kidney stone, as reported in the literature [9]. Osteoporosis is a risk factor for bone fracture even in the case of minor trauma, which is attributed to high-energy shock wave lithotripsy [9]. In our case, following ESWL, a vertebral transverse process fracture occurred in a patient who was not known to have any medical illness.

A transverse process fracture of the lumbar vertebra is usually trauma-related, but no history of trauma was present in our case. Following ESWL, back or flank pain is generally considered to be related to the remaining of the fragmented stone or an ESWL renal-related complication, which is the most common presentation. However, a vertebral fracture could be one of the differential diagnoses. Imaging plays a central role in the diagnosis of ESWL-related complications; for better delineation, CT is indicated [14].

We propose that, during ESWL sessions, any movement of the patient, either intentional or breathing-related, might lead to a change in stone localization that may result in non-focused stone-based shock wave delivery. This may explain the occurrence seen in the present case. A transverse process fracture is considered a stable fracture, which needs bed rest and proper analgesia.

The number of ESWL sessions and timing are controversial. As per American Urological Association guidelines, if the initial ESWL fails, a urologist should offer endoscopic therapy next. This statement is not addressed by the European Association of Urology (EUA). In our case, the patient had an initial partial response, and the decision to have other sessions of ESWL was a shared decision between the physician and the patient. The patient elected to have repeated sessions of ESWL as he had a partial response. The number of ESWL sessions for patients with a partial response is also controversial. In one study that involved 122 patients, 69.7% of patients became completely stone-free after a maximum of four sessions of ESWL [15]. The timing of ESWL sessions is also controversial. According to the EUA guidelines, there are no conclusive data on the intervals required between ESWL sessions. However, clinical experience indicates that repeat sessions are feasible (within one day for ureteral stone). In our case, the timing and number of sessions were mainly affected by patient preference.

## Conclusions

Proper positioning of shock waves to target stones, throughout the ESWL sessions, is a highly important step to maximize stone fragmentation rate and lower complications, possibly caused by non-focusing waves. However, patient movement may result in scattered shock waves to surrounding tissues, which may affect vertebrae. Irrespective of whether bone diseases are a risk factor for ESWL-related fractures, ESWL-related skeletal fractures need to be investigated more in the future. Other factors that may increase the risk of such complications may include the stone HU and the number of ESWL sessions. This also needs to be investigated and addressed in the future.
